# A Review of the Toxicity of Ingredients in e-Cigarettes, Including Those Ingredients Having the FDA’s “Generally Recognized as Safe (GRAS)” Regulatory Status for Use in Food

**DOI:** 10.1093/ntr/ntae123

**Published:** 2024-05-24

**Authors:** Nada O F Kassem, Robert M Strongin, Andrea M Stroup, Marielle C Brinkman, Ahmad El-Hellani, Hanno C Erythropel, Arash Etemadi, Vernat Exil, Maciej L Goniewicz, Noura O Kassem, Theodore P Klupinski, Sandy Liles, Thivanka Muthumalage, Alexandra Noël, David H Peyton, Qixin Wang, Irfan Rahman, Luis G Valerio

**Affiliations:** Health Promotion and Behavioral Science, San Diego State University, San Diego, CA, USA; Hookah Tobacco Research Center, San Diego State University Research Foundation, San Diego, CA, USA; Department of Chemistry, Portland State University, Portland, OR, USA; Behavioral Health and Health Policy Practice, Westat, Rockville, MD, USA; College of Public Health, The Ohio State University, Columbus, OH, USA; Center for Tobacco Research, The Ohio State University Comprehensive Cancer Center, Columbus, OH, USA; Center for Tobacco Research, The Ohio State University Comprehensive Cancer Center, Columbus, OH, USA; Division of Environmental Health Sciences, College of Public Health, The Ohio State University, Columbus, OH, USA; Department of Chemical and Environmental Engineering, Yale University, New Haven, CT, USA; Department of Psychiatry, Yale Center for the Study of Tobacco Products (YCSTP), Yale School of Medicine, New Haven, CT, USA; Metabolic Epidemiology Branch, Division of Cancer Epidemiology and Genetics, National Cancer Institute, NIH, Bethesda, MD, USA; School of Medicine, St. Louis University, St. Louis, MO, USA; Department of Health Behavior, Roswell Park Comprehensive Cancer Center, Buffalo, NY, USA; Health Promotion and Behavioral Science, San Diego State University, San Diego, CA, USA; Hookah Tobacco Research Center, San Diego State University Research Foundation, San Diego, CA, USA; Battelle, Columbus, OH, USA; Health Promotion and Behavioral Science, San Diego State University, San Diego, CA, USA; Hookah Tobacco Research Center, San Diego State University Research Foundation, San Diego, CA, USA; School of Health Sciences, Purdue University, West Lafayette, IN, USA; Comparative Biomedical Sciences, School of Veterinary Medicine, Louisiana State University, Baton Rouge, LA, USA; Department of Chemistry, Portland State University, Portland, OR, USA; Department of Environmental Medicine, University of Rochester Medical Center, Rochester, NY, USA; Department of Environmental Medicine, University of Rochester Medical Center, Rochester, NY, USA; Division of Nonclinical Science (DNCS), Office of Science/Center for Tobacco Products, U.S. Food and Drug Administration, Silver Spring, MD, USA

## Abstract

Some firms and marketers of electronic cigarettes (e-cigarettes; a type of electronic nicotine delivery system (ENDS)) and refill liquids (e-liquids) have made claims about the safety of ingredients used in their products based on the term “GRAS or Generally Recognized As Safe” (GRAS). However, GRAS is a provision within the definition of a food additive under section 201(s) (21 U.S.C. 321(s)) of the U.S. Federal Food Drug and Cosmetic Act (FD&C Act). Food additives and GRAS substances are by the FD&C Act definition intended for use in food, thus safety is based on oral consumption; the term GRAS cannot serve as an indicator of the toxicity of e-cigarette ingredients when aerosolized and inhaled (ie, vaped). There is no legal or scientific support for labeling e-cigarette product ingredients as “GRAS.” This review discusses our concerns with the GRAS provision being applied to e-cigarette products and provides examples of chemical compounds that have been used as food ingredients but have been shown to lead to adverse health effects when inhaled. The review provides scientific insight into the toxicological evaluation of e-liquid ingredients and their aerosols to help determine the potential respiratory risks associated with their use in e-cigarettes.

**Implications**

The rise in prevalence of e-cigarette use and emerging evidence of adverse effects, particularly on lung health, warrant assessing all aspects of e-cigarette toxicity. One development is manufacturers’ stated or implied claims of the safety of using e-cigarette products containing ingredients determined to be “Generally Recognized As Safe” (GRAS) for use in food. Such claims, typically placed on e-cigarette product labels and used in marketing, are unfounded, as pointed out by the United States Food and Drug Administration (FDA)^1^ and the Flavor and Extract Manufacturers Association (FEMA).^2^ Assessment of inhalation health risks of all ingredients used in e-liquids, including those claimed to be GRAS, is warranted.

Electronic cigarettes (e-cigarettes; a type of electronic nicotine delivery system (ENDS)) are devices that generate inhalable aerosols by electrically heating solutions (e-liquids) typically containing the solvents propylene glycol (PG) and glycerol (GL), various concentrations of nicotine, organic acids in the case of nicotine salt formulations, flavoring chemicals (flavorants), and sometimes other ingredients such as color additives, vitamins, and natural and synthetic sweeteners.^3,4^ E-cigarette use, often referred to as “vaping,” has become prevalent among smokers and nonsmokers across the globe.^5,6^

The global increase in the popularity of vaping, particularly among youth, is a serious public health problem.^5^ In the United States (U.S.), during 2020–2021, the U.S. Centers for Disease Control and Prevention (CDC) via the National Health Interview Survey (NHIS) estimated that the prevalence of current e-cigarette use among U.S. adults (≥18 years) increased significantly from 3.7% (9.1 million) to 4.5% (11.1 million), with the highest rate (9.4% in 2020 and 11% in 2021) reported by young adults (18–24 years).^7,8^ The 2022 U.S. National Youth Tobacco Survey (NYTS) demonstrated that e-cigarettes were the most commonly used tobacco product among high school and middle school students.^9^ The 2022 NYTS data indicated that 28.9% (4.41 million) of high school students and 8.5% (1.01 million) of middle school students (overall, 5.45 million) had ever used e-cigarettes.^10^ Furthermore, 14.1% (2.14 million) of high school students and 3.3% (380,000) of middle school students (overall, 2.55 million) reported current e-cigarette use,^9^ of whom 84.9% used flavored e-cigarettes; the most commonly used flavor type was fruit (69.1%), followed by candy, desserts, and other sweets (38.3%), mint (29.4%), and menthol (26.6%).^11^ Nationally representative studies document that flavors are consistently the top reason youth use ENDS.^12,13^

The perception of e-cigarettes as reduced- or low-harm products significantly predicted adolescent and young adult initiation of their use^14^ despite growing evidence that e-cigarettes are not risk-free.^3,15,16^ While long-term adverse health effects of using e-cigarettes are not yet known,^3^ their use has been associated with lung inflammation and oxidative stress, deoxyribonucleic acid (DNA) damage, endothelial dysfunction, arterial stiffness, and susceptibility to cardiovascular and pulmonary diseases.^15,17–19^ Emerging evidence of possible adverse short-term health effects of e-cigarettes, particularly on lung health, and the unknown health effects from long-term use, warrant the assessment of all aspects of their toxicity.^5,16^

Some companies manufacture, market, and sell e-cigarettes and refill liquids (e-liquids) containing ingredients that they label as “Generally Recognized As Safe” (GRAS). However, GRAS is a provision within the definition of a food additive from sections 201(s) of the U.S. Federal Food Drug and Cosmetic Act (FD&C Act) that states that “any substance that is intentionally added to food is a food additive that is subject to premarket review and approval by U.S. Food and Drug Administration (FDA), unless the substance is generally recognized, among qualified experts, as having been adequately shown to be safe under the conditions of its intended use, or unless the use of the substance is otherwise excepted from the definition of a food additive.”^20^ Hereafter, the term GRAS appears in quotes (ie, “GRAS”) to designate instances in which the term has been inappropriately used to label tobacco products.

The Flavor and Extract Manufacturers Association (FEMA), a national association of the U.S. flavor industry, performs GRAS determinations of flavor ingredients independent of the FDA and has clarified that the FEMA GRAS program assesses only the safety of food ingredients that are ingested and not ingredients that are inhaled.^2^Figure 1 is an example of how manufacturers use the “GRAS” term to inform the general public that “all chemicals” contained in the e-liquid are supposedly “generally recognized as safe by the FDA for human ingestion.”^21^ This labeling statement is problematic in several ways: (1) Some ingredients of e-liquids, such as nicotine, have not been concluded to be GRAS for use in food by FDA or FEMA—this labeling indicates that all ingredients are “GRAS”; (2) Using the “GRAS” term on labels or in marketing for e-cigarette products may lead to a misperception that the products are safe; and (3) By definition in the FD&C Act, the GRAS provision applies only to ingredients added to food.

**Figure 1.**
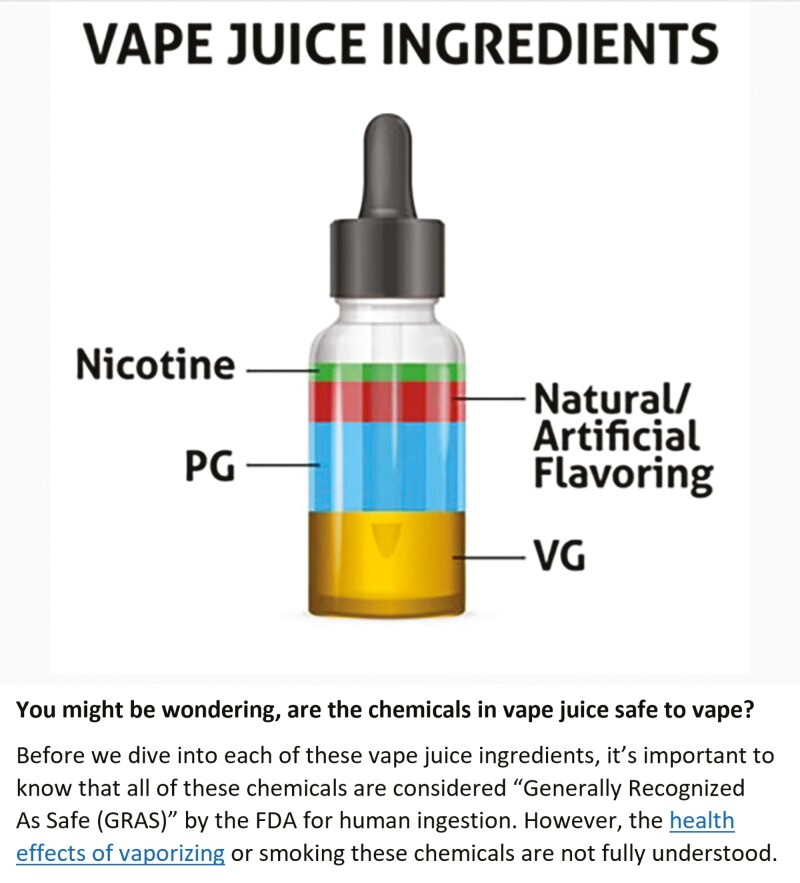
Use of the GRAS term for e-liquid ingredients.^21^

Exposure pathway (eg, dermal, inhalation, ingestion) is a critical component in determining the toxicity of a chemical compound. Evidence of inhalation toxicity should be examined when determining the safety of e-liquid ingredients and corresponding emissions. However, current data are limited regarding the potential adverse acute and chronic health effects of inhaling aerosols of the e-liquid ingredients, either alone or in combination, and regarding how frequency of use may contribute to those effects.

This review discusses our concerns with the GRAS provision being applied to e-cigarette products and provides examples of chemical compounds that have been used as food ingredients but have been shown to lead to adverse health effects when inhaled. The review provides scientific insight into the toxicological evaluation of e-liquid ingredients and their aerosols to help determine the potential respiratory risks associated with their use in e-cigarettes.

## The Generally Recognized As Safe Provision

GRAS is a provision within the definition of a food additive in the FD&C Act. As stated in FDA’s GRAS Final Rule (81 FR 54960), under section 201(s) of the FD&C Act, it is the use of a substance, rather than the substance itself, that is eligible for GRAS status.^22^ Per the Final Rule, “General recognition of safety requires common knowledge, throughout the expert scientific community knowledgeable about the safety of substances directly or indirectly added to food, that there is a reasonable certainty that the substance is not harmful under the conditions of its intended use.”^22^ In addition, the FDA has published guidance about the regulatory framework for substances intended for use in human food on the basis of the GRAS provision and has information available about the history of GRAS.^20,23^ Some of the different ways in which substances may be given the GRAS provision are described in Table 1.

**Table 1.** Brief History of Processes by Which Substances may be Given the GRAS Provision^20^

Context	General description of process
Food ingredients in use before 1958	Conditions for the assumption of GRAS are outlined in §182 of 21 CFR for those food ingredients that were in use before 1958 “without known detrimental effects.”^24^ Not all applicable ingredients are specifically listed.
Substances given the GRAS provision from 1958 to 1962	Numerous GRAS substances that typically did not receive detailed scientific review are listed in §182 of 21 CFR.^24^ They include synthetic flavoring substances and adjuvants, several of which happen to be used in e-liquids (eg, cinnamaldehyde, ethyl butyrate, limonene). Some of the other types of GRAS substances listed in §182 include natural extracts, spices, essential oils, oleoresins, and stabilizers.
Substances assessed for the GRAS provision after 1962	*GRAS Affirmation* Some substances that had originally been assumed to qualify for the GRAS provision were subsequently affirmed to merit the GRAS provision via formal testing and are listed in 21 CFR in §184 (direct food substances) or §186 (indirect food substances).^24^ Some of the formal testing results are products of the Select Committee on GRAS Substances (SCOGS), which was established in the 1970s and consisted of independent consultants who reviewed and evaluated the available information on GRAS substances. After receiving a final SCOGS report, the FDA scientifically reviewed the submitted documents and, if appropriate, issued a final rule affirming the GRAS provision for a given substance.
	*GRAS Notification* Since 1997, the FDA has provided a voluntary mechanism to determine the GRAS provision for a substance evaluated by a non-government entity (eg, a manufacturer or an independent panel of experts) in accordance with the FDA-specified GRAS notification procedure.^101^ If the evaluation leads to the determination of GRAS for a substance under conditions of its intended use, the entity may voluntarily choose to submit a notice for FDA review.
	If the entity chooses to submit a GRAS notice for FDA review, the GRAS notification should include—according to current practices—a clear description of the substance, the applicable conditions of use, and the basis for the GRAS determination, which can be either scientific procedures or common use in food.^102^ The FDA is then supposed to evaluate the submitted notice and to respond to the notifier with one of three possible responses: ◦ The FDA does not question the basis for the GRAS determination; *or* ◦ The notice does not provide a sufficient basis for a GRAS determination (eg, the notice lacks appropriate data and information, or the available information raises questions about the safety of the substance); *or* ◦ The FDA is no longer evaluating the GRAS notice at the notifier’s request.
	The FDA maintains a webpage with a readily searchable inventory of GRAS notices that have been filed since 1998.^103^
	If the entity chooses not to submit a GRAS notice for FDA review for a case in which the applicable testing results led to the assignment of the GRAS provision for a substance, the GRAS provision is valid. Because of this possibility, there are numerous GRAS chemicals that are not explicitly included on the FDA’s lists of GRAS substances, including many flavorants that were assessed through the commonly used FEMA GRAS program.^28^ The FEMA web site includes a page that can be used to search for the GRAS provision of specific chemicals, reflecting results from the FEMA GRAS program when applicable.^104^

The information in this table is not intended to serve as guidance for legal or regulatory purposes. References 101 to 104 are included in the Supplementary Material.

In 21 Code of Federal Regulations (CFR) 170.3(m), food is defined as “human food, substances migrating to food from food-contact articles, pet food, and animal feed.”^24^ Therefore, the regulations and guidance are clear that ENDS products are not included in the legal definition of food or categories of food. Since GRAS only applies to substances intended to be directly added to food with an estimated dietary exposure, technical effect in food, and specific use levels to certain food categories, it is not possible to extrapolate the “intended conditions of use” of the GRAS substance to an ingredient added to and used in e-cigarettes. E-cigarettes are tobacco products, not food, and thus are regulated differently. Heating and aerosolizing the e-liquid in an e-cigarette may alter the level of exposure to any substance in the e-liquid and may result in exposure to any degradation products of that substance.

## GRAS Substances and Adverse Effects via Inhalation

Clapp et al. (2020) suggested amending Paracelsus’ dictum of the “dose makes the poison” to also include “the route of exposure makes the poison.”^25^ Many ingested substances, for example, are broken down by metabolizing and detoxifying enzymes in the gastrointestinal tract and liver. In the case of inhalation, although the lungs possess different metabolic and defense mechanisms, it is generally unclear how and to what extent sensitive lung tissue is negatively impacted by vaping-related exposures.

The inhalation of e-cigarette aerosol exposes lung tissue to multiple chemicals including nicotine, PG, GL, and flavorants. Inhalation exposures to these chemicals are not part of the criteria used by the FEMA expert panel in the GRAS safety evaluation process, meaning that chemicals on FEMA’s GRAS list have not been assessed for potential respiratory toxicity, and certainly not for addition to e-cigarettes.^26–28^

Vitamin E acetate (21 CFR 182.8892 α-Tocopherol acetate) is an example of an important nutrient and a common food additive that is listed as GRAS but was linked to lung injuries in the 2019–2020 U.S. EVALI (e-cigarette, or vaping, product use-associated lung injury) outbreak.^29^ Amid the outbreak, the FDA and CDC found an association between EVALI and vitamin E acetate exposure, due to its use as a diluent in some THC-containing vaped products.^29^ Although the specific cause of EVALI was not established, analysis of patient samples implicated vitamin E acetate as having a role in lung damage.^30^ This is an example of how a GRAS ingredient could become harmful when exposure occurs outside of the intended conditions of use (ie, inhaled not ingested).

## GRAS Flavors in e-Cigarette Products

National data show that in 2022, 2.55 million U.S. middle and high school students reported current e-cigarette use, of whom most (84.7% and 84.9%, respectively) reported using the flavored varieties (fruit; candy, desserts, or other sweets; mint; and menthol).^11,31,32^ The availability of flavored products is a primary reason for using e-cigarettes, particularly among youth.^3,33^ E-liquids for disposable and refillable e-cigarettes are available in numerous flavors.^3^ These products often contain a variety of flavorants, yet for most, inhalation safety has not been determined. At present, firms may choose which ingredients they believe are appropriate; however, the scientific review of premarket tobacco product applications includes an evaluation of the product formulation and potential health risks of inhaled flavorants.^34^

While several of the flavorants used in e-liquids may be GRAS for use as flavors in food, a reference to their GRAS regulatory status does not contribute to understanding the health risks of e-cigarette products. GRAS regulatory status as an ingredient added to food cannot be used to determine or characterize the toxicological profile of e-liquid flavorants or other ingredients upon inhalation from e-cigarettes. Referring to GRAS regulations or other GRAS conclusions such as performed by FEMA to establish a risk profile or characterize the potential toxicity of e-cigarette use is inappropriate because e-cigarettes are not meant to be ingested. In fact, some flavored e-cigarettes have been reported to induce more toxic effects than the nonflavored type in cell culture (non-inhalation model) and mice studies (inhalation model).^35,36^ Some common e-liquid flavorants that are GRAS by regulation or other GRAS conclusions for oral ingestion via addition to food but are known to be associated with inhalation toxicity include benzaldehyde (cherry/almond flavor), cinnamaldehyde (cinnamon flavor), ethyl vanillin (vanilla flavor), and diacetyl (buttery flavor).^37–40^ Benzaldehyde, for example, has been shown to serve as a precursor to benzene when used in some e-cigarettes, and benzene is one of FDA’s published Harmful and Potentially Harmful Constituents (HPHCs) in tobacco products and tobacco smoke.^41,42^

Table 2 contains more examples and detailed information on chemicals that are listed in FDA’s GRAS regulations for use in food,^24^ yet are potentially hazardous when used in e-cigarettes. One substance in Table 2, diacetyl, is the established causative agent of bronchiolitis obliterans (popcorn lung) upon inhalation, yet a safe compound when ingested.^43,44^ Another is triacetin, which is used to enhance the flavor and/or physical properties of food formulations and has been found to catalyze the formation of aldehyde toxicants.^45^ Lipids and oils, such as decanal, should never be used in a product intended for inhalation without rigorous safety testing, since such compounds are widely known to cause lipoid pneumonia upon inhalation.^46^ Overall, the substances listed in Table 2 can potentially cause significant harm when used in e-cigarettes via proinflammatory effects as well as immune suppression, respiratory tract irritation, and cytotoxicity.

**Table 2.** Substances Listed in FDA’s GRAS Regulations and Potential Respiratory Health Risks When Used in e-Cigarettes

GRAS substance ^	21 CFR GRAS Regulation #	Risks associated with inhalation^†^ or cell exposure^‡^ when used in e-cigarettes	Study design
Acetaldehyde	§182.60	^†^Nasopharyngeal & laryngeal carcinogenesis observed in rats.^105^ Listed on FDA’s established list of HPHCs as a carcinogen.^42^	Wistar rats exposed to 0–3000 ppm chemicals for 28 months.^105^
Acetoin	§182.60	^†^Acetoin is a precursor to diacetyl, which induces respiratory impairment and obliterative bronchiolitis when aerosolized and inhaled.^43,44,93^ Acetoin is on the list of proposed additions to FDA’s HPHCs as a respiratory toxicant.^106^	C57BL/6 mice exposed to 100 ppm for 12 weeks.^44^
Cinnamaldehyde	§182.60	^‡^Rapidly reacts with PG & GL to form *cinnamaldehyde PG (or GL) acetals.*^90^ Aerosolized cinnamaldehyde (with PG) caused death of lung epithelium.^107^ PG is on the list of proposed additions to FDA’s HPHCs as a respiratory toxicant.^106^ GL is on the list of proposed additions to FDA’s HPHCs as a carcinogen.^106^	A549 cells exposed to 0–6 total puff equivalents of the chemicals for 48 h.^107^
Diacetyl	§184.1278	^†^Aerosolized diacetyl showed dose-dependent pulmonary toxicity with mild nasal and peri-bronchial inflammation in mice,^44^ as the respiratory impairment and obliterative bronchiolitis.^43,44^ Diacetyl is on the list of proposed additions to FDA’s HPHCs as a respiratory toxicant.^106^	C57BL/6 mice exposed to 100 ppm chemicals for 12 weeks.^44^
Ethyl acetate	§182.60	^†^Throat irritant and a high dosage (>400 ppm) could cause dizziness or loss of consciousness.^108^ Ethyl acetate is on the list of proposed additions to FDA’s HPHCs as a respiratory toxicant.^106^	OSHA 8-h total weight average (TWA) permissible exposure limit (PEL): 400 ppm.^109^
Eugenol	§184.1257	^†^Irritation and abnormal breathing in rats.^110^	SD rats exposed to 0–2.58 g/L chemicals for 14 days.^110^
Tocopherol acetate	§182.8892	^†^Associated with lung injury and associated with the 2019–2020 outbreak of EVALI in the United States.^111^	Major chemical linked with EVALI patient.^111^
Triacetin	§184.1901	Promotes the formation of toxicants such as acrolein and acetaldehyde from PG and GL.^45^	No toxicity test available currently.
Linalool	§182.60	^†^Promotes free radical formation^112^ and can act as an allergen and irritant.^113^ ^‡^Free radical releasing; inhibited cytokine expression from activated THP-1 cells.^114^	Human patient exposed to 0.8–4.4 mg/cm^2^ by patch for 9 months;^113^ THP1 cells exposed to 1000 μM chemicals for 24 h.^114^
Benzaldehyde	§182.60	^‡^Rapidly reacts with PG & GL to form *benzaldehyde PG (or GL) acetals,*^90^ which are respiratory tract irritants; cellular toxicity in lung epithelium; inhibits mitochondrial functions.^94^	BEAS-2B and A549 exposed to 1–10 mM chemicals for 24 h.^94^
Carvone	§182.60	^‡^Cytokine inhibitions indicating potential immune suppression.^114^	THP1 cells exposed to 1 mM chemical for 24.^114^
Decanal	§182.60	^‡^Cytotoxicity in lung epithelium and monocytes with decreased viability; potential immune suppression.^114^	THP1 and BEAS-2B cells exposed to 1 mM chemical for 24 h.^114^
Ethyl butyrate	§182.60	^‡^Activated inflammatory response in naïve monocytes, with increased IL-6 and IL-8.^114^	THP1 cells exposed to 1 mM chemical for 24 h.^114^
Ethyl vanillin	§182.60	^‡^Rapidly reacts with PG & GL to form *ethyl vanillin PG (or GL) acetals.*^90^ Promotes free radical formation.^112^ Cytotoxicity in lung epithelium cells; obstructive or restrictive lung diseases.^115^	BEAS-2B cells exposed to 0–10 mg/mL chemicals for 48 h.^115^
Limonene	§182.60	^‡^Decreased cell viability and increased inflammatory response in naïve monocyte; potential immune repression effects.^114^	THP1 cells exposed to 1 mM chemical for 24 h.^114^

References 105 to 115 are included in the Supplementary Material.

^GRAS substance demonstrated to be safe via the oral route for use in food.^24^

^†^Experimental models with aerosol exposure.

^‡^Direct exposure to the cells via diluted e-liquid.

An analysis of the thermal degradation of the e-cigarette flavorants cinnamaldehyde, vanillin, eugenol, and menthol (all listed in FDA’s GRAS regulations for flavoring use) showed that, depending on the operating temperature of the e-cigarette, these flavorants could lead to significant increases in emitted levels of toxicants in aerosol such as formaldehyde, acetaldehyde, and benzene.^47^ These observations are further evidence that the safety of use implied by the GRAS term cannot be extrapolated to inhalation use. In addition, compounds listed as GRAS in FDA’s regulations or concluded to be GRAS by FEMA, can undergo pyrolysis and pyrosynthesis in e-cigarettes, leading to potentially problematic breakdown products.^48^ Generally, the extent of thermal degradation of flavorants depends on device parameters such as resistance, voltage output, resulting power output, and wicking efficiency, as well as user puffing behavior such as flow rate and puff length. Taken together, the inhalation health risks shown for these substances present a sharp contrast to the relatively innocuous properties that these substances have under conditions of intended use in food products.

## Other GRAS Substances Used as Solvents in e-Cigarettes

In addition to numerous flavorants and additives used in e-cigarettes, the solvents PG and GL, regulated as GRAS for use in food (21 CFR 184.1666 and 21 CFR 182.1320, respectively), are the major e-liquid constituents. Several studies have shown that heating PG and GL during e-cigarette use can result in their partial thermal degradation to HPHCs and toxicants such as formaldehyde, acetaldehyde, glycolaldehyde, hydroxyaldehyde, glyoxal, propanal, and acrolein.^49–52^ The extent of the thermal degradation of PG and GL depends on the operating parameters of the e-cigarette.^53,54^

PG is easily absorbed by oral and dermal routes and metabolized into lactic acid, among other chemicals.^55^ While the exposure limits are unclear, the Occupational Safety and Health Administration (OSHA) has established an 8-hour threshold limit of 3.2 ppm or 10 mg/m^3^.^56^ In e-cigarette users, these levels may be exceeded depending on vaping frequency and puffing topography. Inhalation of PG aerosols by humans has been associated with irritation of the respiratory airways and throat.^57^ In addition, PG aerosol has been identified by the Agency for Toxic Substances and Disease Registry (ATSDR) as having potential adverse respiratory effects.^58^ While PG is considered safe as a food ingredient with a dietary limit of 25 mg/kg body weight for humans (Lethal Dose (LD)_50_ of 20 g/kg body weight in rodents),^59,60^ its cardiopulmonary safety is ambiguous, as respiratory allergic responses, alterations in goblet cells, and adverse hemodynamic effects have been observed in acute toxicity studies in animals.^61,62^

GL has an exposure limit of 15 mg/m^3^ by OSHA more than an 8-h time-weighted average,^56^ and is considered safe for consumption orally (LD_50_ of approximately 2.53–37.7 g/kg body weight in rodents).^63^ Inhalation studies in animals have shown squamous metaplasia effects with a no-observed-adverse-effect-level of 0.167 mg/L in rodents.^63^ Additional recent studies have shown that exposure to e-cigarette solvents increases urinary acetate levels associated with pulmonary irritant responses and endothelial dysfunction.^61^

Unexpectedly, in vivo inhalation of PG and GL, and not nicotine, can affect the expression of circadian molecular clock genes that affect the circadian rhythmicity.^62^ The physicochemical properties of PG and GL also impact aerosol particle size and distribution in the respiratory tract.^64^ Further, PG and GL can exhibit molecular interactions with lung surfactant proteins and lipids such as phosphatidylcholines. Based on the current understanding of the acute and chronic toxicity of reactive carbonyl compounds and oxidative damage promoted by PG and GL during e-cigarette use, the inhalation safety of vaping PG and GL must be re-evaluated using target organ toxicological and pharmacokinetic studies.

## Interaction of GRAS Ingredients With Nicotine

In puffing machine-generated e-cigarette aerosols, nicotine yield is influenced by device type (eg, tank style vs. mod/pod), operational settings (eg, power output), user vaping topography (eg, puff duration and frequency), ratio of nicotine form, flavorant presence, and e-liquid solvent ratio (eg, PG/GL).^65,66^ PG and GL, while listed as GRAS in food under conditions of their intended use, are documented to interact with nicotine to impact the physical properties of e-cigarette aerosols such as particle size, and consequently the depth of pulmonary deposition and interactions of the aerosol components with the respiratory tract cells.^67^

In e-liquids, the ratio of unprotonated free-base nicotine to protonated nicotine is variable and determined by the pH, which in turn is defined by the amount and nature of organic acid used as counterion, the ratio of PG and GL, and the presence of flavoring chemicals.^66,68–71^ E-liquids with a PG content >70% aerosolize faster and lead to greater nicotine concentration in the e-cigarette aerosol than e-liquids with higher proportions of GL.^72^ This was further confirmed in e-cigarette users where e-liquids with higher contents of PG were associated with increased nicotine delivery.^73,74^

## Toxicity of Other Food Ingredients

In addition to solvents and flavor substances regulated by FDA as GRAS for use in food,^75^ there are other ingredients used in food that are sometimes used in e-cigarette products, such as color additives, vitamins, and sweeteners. These compounds have received relatively little attention in the context of their use in e-cigarette products.

### Color Additives

Colored e-liquids and aerosols augment the sensory appeal of flavored e-cigarettes; however, we are aware of only one peer-reviewed study focused on the determination of synthetic food dye ingredients in e-cigarettes. The researchers found Allura Red AC and Brilliant Blue FCF in red and blue e-liquids and the pair Tartrazine and Brilliant Blue FCF in green e-liquids.^76^ Concentration of the dyes were relatively low and a user would have to consume e-liquids on the scale of kg/day to exceed the acceptable daily intake (ADI) thresholds set by the Joint Expert Committee on Food Additives (JECFA).^76,77^

However, the ADI is generally based on oral ingestion and determined levels are thus *not* based on toxicological inhalation data. In addition, ADI levels do not consider possible thermal breakdown products, such as carcinogenic amines in the case of dye molecules, which may form during storage and/or during heating/aerosolizing. Moreover, commercial samples of Allura Red AC, Sunset Yellow FCF, and Tartrazine had previously been found to contain residual benzidine, a human carcinogen.^78^ Tartrazine is also known to cause severe allergic reactions.^79^

### Vitamins

Vitamin-containing e-cigarettes comprise another class of products for which inhalation toxicity has been under-investigated. Commercial e-liquids with vitamin additives are reported to be widely available either with or without nicotine.^80^ These products typically contain vitamin B12, but may also include other vitamins such as A, C, and E. Marketing claims have stated that vaping enhances vitamin absorption and leads to increased energy.^80^ Some products additionally contain ingredients such as essential oils, theanine, and green tea extract.^80^ Manufacturers’ claims that these products are effective against cancer, asthma, and attention-deficit/hyperactivity disorder, and can prevent anemia,^81^ have prompted warning letters from the FDA.^82^ Due to the health claims, the FDA has stated that at least some of these products are considered unapproved new drugs.^83^ While vitamins are essential nutrients reasonably expected to be safe for ingestion under established daily values, evidence-based research on the inhalation effects of these often marketed as “wellness” products is lacking.

### Sweeteners

Sugars are natural components of tobacco. Historically, tobacco companies have added glucose, fructose, and sucrose, at up to 4% of a cigarette’s weight, to enhance tobacco flavor and reduce harshness.^84^ During vaping, e-liquids achieve temperatures at which sugars will degrade to form toxicants including aldehydes and furans.^85,86^ Sweeteners, such as sucralose (eg, Splenda), have been recently reported to transfer from e-liquids into the aerosol using low-power e-cigarettes.^87^ In the vaping process, sucralose enhances toxicant formation,^88^ including the formation of two chloropropanols that are classified as IARC group 2B possible human carcinogens.^89^

## Storage of e-Cigarettes With GRAS Ingredients

Studies of potential e-liquid toxicity generally begin with the assumption that an e-liquid is simply the sum of its ingredients. However, it has been established that aldehyde flavorants such as benzaldehyde, cinnamaldehyde, or vanillin, can react with the common e-liquid solvents PG and GL to form stable acetals within hours.^90–92^ Furthermore, other chemicals are known to react during e-liquid aging, including acetoin, which can be oxidized to yield diacetyl.^93^

Evidence of potential negative health effects from these reactions is emerging,^94^ and transfer of the products to the aerosol, resulting in user exposure to these toxicants, has been demonstrated.^90,91^ This underscores the importance of characterizing constituents not only in the e-liquid, but even more critically, in the e-liquid aerosol to which e-cigarette users are exposed. Only the testing of generated aerosol in a range of devices can result in a meaningful understanding of the potential health effects of the e-liquid in question.^90^

## Marketing Tactics Using the “GRAS” Term

Some firms and marketers of e-cigarettes may make unfounded claims about the ingredients of their product to imply a certain safety of vaping. For example, some web sites aver ingredient safety by claiming “GRAS” status, and thus imply overall product safety, by listing their e-cigarette product ingredients alongside examples of food products that contain the same ingredients, eg, “salad dressing, ice cream, soft drinks, packaged frosting, boxed cake mix, and commercial food coloring,”^21,95^ thereby obscuring the different route of exposure: inhalation for e-cigarettes, ingestion for food products.

These marketing tactics can lead to misperceptions about the risks of vaping. To curb this misinformation, the FDA Center for Tobacco Products (CTP) Premarket Tobacco Product Applications for Electronic Nicotine Delivery Systems (Revised) Guidance to the industry discussed the term GRAS: “e-liquid is not food and is not intended for oral ingestion; therefore, the fact that some substances have been designated as GRAS for food does not mean that they are safe for inhalation.”^1^ This clarity is a good first step and could be strengthened by a public information campaign with the same message. To protect public health, FDA closely monitors industry compliance with the Tobacco Control Act and may take action when violations occur.^96^

## Perception of the “GRAS” Term in e-Cigarettes

Claims of “GRAS” on the labels of e-cigarette packaging can result in unfounded and incorrect perceptions of the safety of these products. As laid out in the previous sections, the GRAS provision within the definition of a food additive from the FD&C Act, does not apply to inhaled e-cigarette aerosol, and only applies to use of a substance in human or animal food “under the conditions of its intended use.” Additionally, the FDA does not currently require that e-liquid and e-cigarette packaging include a comprehensive list of ingredients present in the e-liquid or aerosol including potential flavorants,^97,98^ adding to the uncertainty around relative safety.

In the only known study that assessed perceptions of e-cigarette flavoring safety related to the understanding of the “GRAS” term, 80% of U.S. college students surveyed (*N* = 567) agreed that e-cigarette flavorings are “generally recognized as safe” by the FDA.^99^ Among participants who considered flavorings to be “GRAS,” the majority thought that a GRAS substance was safe to ingest *and* inhale; only 22% of respondents knew that the “GRAS” term was only related to ingestion.^99^ Such misconceptions may be the result of incorrect e-liquid advertisements or web site claims. Also, employees at vape shops may be sharing misinformation about the ingredients in e-liquids.^100^

## Conclusions

In conclusion, knowledge about the inhalation toxicity of individual e-cigarette ingredients (independent of whether a substance may be GRAS for use in food) and mixtures thereof remains limited. Many e-cigarette ingredients, including e-liquid solvents and flavorants, are GRAS for use in food either by FDA regulation or by a FEMA GRAS conclusion, but that conclusion provides no information concerning the toxic potential of these ingredients in contexts other than food consumption. The current lack of relevant inhalation toxicity data is an information gap that complicates regulation as well as the public perception of the health risks of vaping products containing chemicals with or without the GRAS term. Efforts to increase knowledge about the inhalation safety of the numerous chemicals currently present in e-cigarettes would address this gap. Such evidence-based knowledge could empower users of e-cigarette products, the public health community, and the general public to better evaluate the potential risks associated with e-cigarette use.

## Research Suggestions

The issues raised in this review pose challenges and opportunities for researchers and regulators. Based on the findings presented, research that would improve understanding of the risks of e-cigarette use targeting substances that are GRAS for use in food for several e-liquid ingredients as outlined in this manuscript would be important (see Table 3). Additionally, the authors recommend that public health agencies enhance current education campaigns about misleading information that may result in misperceptions of the potential health risks of vaping e-cigarette products containing ingredients incorrectly labeled as “GRAS.”

**Table 3.** Research Suggestions

Suggested Topic Areas for Future Research
• Inhalation toxicity profile of e-liquid ingredients in e-cigarette products, including those regulated as GRAS for use in food. - Toxicity of e-liquid ingredients and their aerosols using varying power profiles and puffing regimes - Interactions between e-liquid ingredients before and after aerosolization to learn of potential formation of toxic byproducts - Toxicity of new chemicals resulting from thermal degradation of e-liquid ingredients • Sources of ambiguity or inaccuracy in marketing strategies, such as use of the GRAS term to imply the safety of e-liquid ingredients in e-cigarette products. - Labels of e-cigarette product packages (eg, GRAS labeling, mislabeling, health claims, ingredient lists, food imagery) - Characteristics of e-cigarette products (eg, comparison of products to common foods) - Online web sites of manufacturers and retailers of e-cigarette products - In-store advertisements for e-cigarette products (eg, vape shops, convenience stores) - Social media advertisements for e-cigarette products (eg, manufacturer and retailer social media) • Appeal and perceived safety of e-liquid ingredients in e-cigarette products, including those ingredients regulated as GRAS for use in food. - Among adolescents and young adults - Among e-cigarette users and non-e-cigarette users

## Supplementary material

Supplementary material is available at *Nicotine and Tobacco Research* online.

ntae123_suppl_Supplementary_Material

## References

[1] FDA. Premarket Tobacco Product Applications for Electronic Nicotine Delivery Systems (Revised) - Guidance for Industry. 2023.

[2] FEMA. Safety Assessment and Regulatory Authority to Use Flavors—Focus on Electronic Nicotine Delivery Systems and Flavored Tobacco Products. 2018; https://www.femaflavor.org/sites/default/files/FEMAGRAS%20Ecig%20092616.pdf

[3] Marques P , Piqueras L, Sanz MJ. An updated overview of e-cigarette impact on human health. Respir Res. 2021;22(1):151. 34006276 10.1186/s12931-021-01737-5 PMC8129966

[4] Breland A , Soule E, Lopez A, et al. Electronic cigarettes: what are they and what do they do? Ann N Y Acad Sci. 2017;1394(1):5–30. 26774031 10.1111/nyas.12977 PMC4947026

[5] Lyzwinski LN , Naslund JA, Miller CJ, Eisenberg MJ. Global youth vaping and respiratory health: epidemiology, interventions, and policies. NPJ Prim Care Respir Med. 2022;32(1):14. 35410990 10.1038/s41533-022-00277-9 PMC9001701

[6] Giovacchini CX , Crotty Alexander LE, Que LG. Electronic cigarettes: a pro-con review of the current literature. J Allergy Clin Immunol Pract. 2022;10(11):2843–2851. 35872217 10.1016/j.jaip.2022.07.009 PMC12922728

[7] Cornelius ME , Loretan CG, Wang TW, Jamal A, Homa DM. Tobacco product use among adults - United States, 2020. MMWR Morb Mortal Wkly Rep. 2022;71(11):397–405. 35298455 10.15585/mmwr.mm7111a1 PMC8942309

[8] Cornelius ME , Loretan CG, Jamal A, et al. Tobacco product use among adults—United States, 2021. MMWR Morb Mortal Wkly Rep. 2023;72(18):475–483. 37141154 10.15585/mmwr.mm7218a1 PMC10168602

[9] Park-Lee E , Ren C, Cooper M, et al. Tobacco product use among middle and high school students—United States, 2022. MMWR Morb Mortal Wkly Rep. 2022;71(45):1429–1435. 36355596 10.15585/mmwr.mm7145a1 PMC9707354

[10] CDC. Tobacco Product Use Among Middle and High School Students—United States, 2022. 2022. 10.15585/mmwr.mm7145a1 PMC9707354 36355596

[11] Cooper M , Park-Lee E, Ren C, et al. Notes from the field: e-cigarette use among middle and high school students—United States, 2022. MMWR Morb Mortal Wkly Rep. 2022;71(40):1283–1285. 36201370 10.15585/mmwr.mm7140a3 PMC9541029

[12] Ambrose BK , Day HR, Rostron B, et al. Flavored tobacco product use among US youth aged 12–17 Years, 2013–2014. JAMA. 2015;314(17):1871–1873. 26502219 10.1001/jama.2015.13802 PMC6467270

[13] Tsai J , Walton K, Coleman BN, et al. Reasons for electronic cigarette use among middle and high school students—National Youth Tobacco Survey, United States, 2016. MMWR Morb Mortal Wkly Rep. 2018;67(6):196–200. 29447148 10.15585/mmwr.mm6706a5 PMC5815490

[14] Li W , Osibogun O, Gautam P, et al. Effect of harm perception on ENDS initiation among US adolescents and young adults: longitudinal findings from the population assessment of tobacco and health (PATH) study, 2013–2018. Drug Alcohol Depend. 2023;244:109784. 36706674 10.1016/j.drugalcdep.2023.109784

[15] Wang L , Wang Y, Chen J, Liu P, Li M. A review of toxicity mechanism studies of electronic cigarettes on respiratory system. Int J Mol Sci . 2022;23(9):5030. 35563421 10.3390/ijms23095030 PMC9102406

[16] CDC. E-Cigarette Use Among Youth and Young Adults. A Report of the Surgeon General. Atlanta (GA). 2016. 30869850

[17] Merecz-Sadowska A , Sitarek P, Zielinska-Blizniewska H, et al. A summary of in vitro and in vivo studies evaluating the impact of e-cigarette exposure on living organisms and the environment. Int J Mol Sci . 2020;21(2):652. 31963832 10.3390/ijms21020652 PMC7013895

[18] Moheimani RS , Bhetraratana M, Yin F, et al. Increased cardiac sympathetic activity and oxidative stress in habitual electronic cigarette users: implications for cardiovascular risk. JAMA Cardiol. 2017;2(3):278–284. 28146259 10.1001/jamacardio.2016.5303 PMC5626008

[19] Singh KP , Lawyer G, Muthumalage T, et al. Systemic biomarkers in electronic cigarette users: implications for noninvasive assessment of vaping-associated pulmonary injuries. ERJ Open Res. 2019;5(4):00182–02019. 10.1183/23120541.00182-2019 PMC6926365 31886159

[20] FDA. FDA’s Approach to the GRAS Provision: A History of Processes. 2018.; https://www.fda.gov/food/generally-recognized-safe-gras/fdas-approach-gras-provision-history-processes. Accessed December 29, 2022.

[21] RuthlessVapor. What is in vape Juice? 2023. https://www.ruthlessvapor.com/blogs/ruthless-e-liquid/whats-in-vape-juice? Accessed April 6, 2023.

[22] FDA. Substances Generally Recognized as Safe. Fed Regist. 2016. https://www.federalregister.gov/documents/2016/08/17/2016-19164/substances-generally-recognized-as-safe

[23] FDA. Guidance for Industry: Regulatory Framework for Substances Intended for Use in Human Food or Animal Food on the Basis of the Generally Recognized as Safe (GRAS) Provision of the Federal Food, Drug, and Cosmetic Act. 2017; https://www.fda.gov/regulatory-information/search-fda-guidance-documents/guidance-industry-regulatory-framework-substances-intended-use-human-food-or-animal-food-basis. Accessed December 14, 2023.

[24] FDA. US Code of Federal Regulations (CFR), Title 21, Chapter I, Subchapter B on Food for Human Consumption. 2022; https://www.ecfr.gov/current/title-21/chapter-I/subchapter-B

[25] Clapp PW , Peden DB, Jaspers I. E-cigarettes, vaping-related pulmonary illnesses, and asthma: A perspective from inhalation toxicologists. J Allergy Clin Immunol. 2020;145(1):97–99. 31715190 10.1016/j.jaci.2019.11.001 PMC7081413

[26] FEMA. Respiratory Health and Safety in the Flavor Manufacturing Workplace. 2012.; https://www.femaflavor.org/sites/default/files/2018-06/FEMA%202012%20Respiratory%20Health%20and%20Safety%20in%20Workplace.pdf

[27] Smith RL , Cohen SM, Doull J, et al; Expert Panel of the Flavor and Extract Manufacturers Association. Criteria for the safety evaluation of flavoring substances. The expert panel of the flavor and extract manufacturers association. Food and Chemical Toxicology: International Journal Published for the British Industrial Biological Research Association. 2005;43(8):1141–1177. 10.1016/j.fct.2004.11.012 15950813

[28] Hallagan JB , Hall RL, Drake J. The GRAS provision—the FEMA GRAS program and the safety and regulation of flavors in the United States. Food Chem Toxicol. 2020;138:111206. 32135216 10.1016/j.fct.2020.111236

[29] Blount BC , Karwowski MP, Shields PG, et al; Lung Injury Response Laboratory Working Group. Vitamin E acetate in bronchoalveolar-lavage fluid associated with EVALI. N Engl J Med. 2020;382(8):697–705. 31860793 10.1056/NEJMoa1916433 PMC7032996

[30] Schupp JC , Prasse A, Erythropel HC. E-cigarettes—operating principle, ingredients, and associated acute lung injury. Pneumologie. 2020;74(2):77–87. 32016924 10.1055/a-1078-8126 PMC7366312

[31] Gentzke AS , Wang TW, Cornelius M, et al. Tobacco product use and associated factors among middle and high school students—National Youth Tobacco Survey, United States, 2021. MMWR Surveill Summ. 2022;71(5):1–29. 10.15585/mmwr.ss7105a1 PMC8923300 35271557

[32] Park-Lee E , Ren C, Sawdey MD, et al. Notes from the field: e-cigarette use among middle and high school students—National Youth Tobacco Survey, United States, 2021. MMWR Morb Mortal Wkly Rep. 2021;70(39):1387–1389. 34591834 10.15585/mmwr.mm7039a4 PMC8486384

[33] Villanti AC , Johnson AL, Ambrose BK, et al. Flavored tobacco product use in youth and adults: findings from the first wave of the PATH Study (2013-2014). Am J Prev Med. 2017;53(2):139–151. 28318902 10.1016/j.amepre.2017.01.026 PMC5522636

[34] FDA. Premarket Tobacco Product Applications and Recordkeeping Requirements. Federal Register 2021; https://www.federalregister.gov/documents/2021/10/05/2021-21011/premarket-tobacco-product-applications-and-recordkeeping-requirements

[35] Lamb T , Muthumalage T, Meehan-Atrash J, Rahman I. Nose-only exposure to cherry- and tobacco-flavored e-cigarettes induced lung inflammation in mice in a sex-dependent manner. Toxics. 2022;10(8):471. 36006150 10.3390/toxics10080471 PMC9413458

[36] Lucas JH , Muthumalage T, Wang Q, et al. E-liquid containing a mixture of coconut, vanilla, and cookie flavors causes cellular senescence and dysregulated repair in pulmonary fibroblasts: implications on premature aging. Front Physiol. 2020;11:924. 33013432 10.3389/fphys.2020.00924 PMC7500211

[37] Behar RZ , Davis B, Wang Y, et al. Identification of toxicants in cinnamon-flavored electronic cigarette refill fluids. Toxicol In Vitro. 2014;28(2):198–208. 24516877 10.1016/j.tiv.2013.10.006

[38] Goniewicz ML , Knysak J, Gawron M, et al. Levels of selected carcinogens and toxicants in vapour from electronic cigarettes. Tob Control. 2014;23(2):133–139. 23467656 10.1136/tobaccocontrol-2012-050859 PMC4154473

[39] Farsalinos KE , Kistler KA, Gillman G, Voudris V. Evaluation of electronic cigarette liquids and aerosol for the presence of selected inhalation toxins. Nicotine Tob Res. 2015;17(2):168–174. 25180080 10.1093/ntr/ntu176 PMC4892705

[40] Bahl V , Lin S, Xu N, et al. Comparison of electronic cigarette refill fluid cytotoxicity using embryonic and adult models. Reprod Toxicol. 2012;34(4):529–537. 22989551 10.1016/j.reprotox.2012.08.001

[41] Pankow JF , Kim K, McWhirter KJ, et al. Benzene formation in electronic cigarettes. PLoS One. 2017;12(3):e0173055. 28273096 10.1371/journal.pone.0173055 PMC5342216

[42] CTP. Harmful and Potentially Harmful Constituents (HPHCs). 2019.

[43] Harber P , Saechao K, Boomus C. Diacetyl-induced lung disease. Toxicol Rev. 2006;25(4):261–272. 17288497 10.2165/00139709-200625040-00006

[44] Morgan DL , Flake GP, Kirby PJ, Palmer SM. Respiratory toxicity of diacetyl in C57BL/6 mice. Toxicol Sci. 2008;103(1):169–180. 18227102 10.1093/toxsci/kfn016 PMC2669658

[45] Vreeke S , Peyton DH, Strongin RM. Triacetin enhances levels of acrolein, formaldehyde hemiacetals, and acetaldehyde in electronic cigarette aerosols. ACS Omega. 2018;3(7):7165–7170. 30087908 10.1021/acsomega.8b00842 PMC6068691

[46] Spickard A, 3rd, Hirschmann JV. Exogenous lipoid pneumonia. Arch Intern Med. 1994;154(6):686–692. 8129503

[47] Kuehl PJ , McDonald JD, Weber DT, et al. Composition of aerosols from thermal degradation of flavors used in ENDS and tobacco products. Inhal Toxicol. 2022;34(11-12):319–328. 35913821 10.1080/08958378.2022.2103602 PMC9830633

[48] El-Hellani A , El-Hage R, Salman R, et al. Electronic cigarettes are chemical reactors: implication to toxicity. Chem Res Toxicol. 2020;33(10):2489–2490. 33021780 10.1021/acs.chemrestox.0c00412 PMC9355289

[49] Ooi BG , Dutta D, Kazipeta K, Chong NS. Influence of the e-cigarette emission profile by the ratio of glycerol to propylene glycol in E-liquid composition. ACS Omega. 2019;4(8):13338–13348. 31460462 10.1021/acsomega.9b01504 PMC6705204

[50] Saliba NA , El Hellani A, Honein E, et al. Surface chemistry of electronic cigarette electrical heating coils: Effects of metal type on propylene glycol thermal decomposition. J Anal Appl Pyrolysis. 2018;134:520–525. 30906089 10.1016/j.jaap.2018.07.019 PMC6428435

[51] Uchiyama S , Ohta K, Inaba Y, Kunugita N. Determination of carbonyl compounds generated from the E-cigarette using coupled silica cartridges impregnated with hydroquinone and 2,4-dinitrophenylhydrazine, followed by high-performance liquid chromatography. Anal Sci. 2013;29(12):1219–1222. 24334991 10.2116/analsci.29.1219

[52] El-Hage R , El-Hellani A, Salman R, et al. Vaped humectants in e-cigarettes are a source of phenols. Chem Res Toxicol. 2020;33(9):2374–2380. 32786548 10.1021/acs.chemrestox.0c00132 PMC9355288

[53] Kosmider L , Sobczak A, Fik M, et al. Carbonyl compounds in electronic cigarette vapors: effects of nicotine solvent and battery output voltage. Nicotine Tob Res. 2014;16(10):1319–1326. 24832759 10.1093/ntr/ntu078 PMC4838028

[54] Talih S , Balhas Z, Eissenberg T, et al. Effects of user puff topography, device voltage, and liquid nicotine concentration on electronic cigarette nicotine yield: measurements and model predictions. Nicotine Tob Res. 2015;17(2):150–157. 25187061 10.1093/ntr/ntu174 PMC4837998

[55] Yu DK , Elmquist WF, Sawchuk RJ. Pharmacokinetics of propylene glycol in humans during multiple dosing regimens. J Pharm Sci. 1985;74(8):876–879. 4032274 10.1002/jps.2600740815

[56] ACGIH. Documentation of the Threshold Limit Values (TLVs) and Biological Exposure Indices (BEIs) - Appendix G. Glycerin mist. 2023; https://www.acgih.org/

[57] NTP. NTP-CERHR Monograph on the Potential Human Reproductive and Developmental Effects of Propylene Glycol. 2004. 15995735

[58] Moline JM , Golden AL, Highland JH, Wilmarth KR, Kao AS. Health effects evaluation of theatrical smoke, haze, and pyrotechnics. 2000.

[59] ATSDR. Toxicological profile for propylene glycol. 1997; https://www.atsdr.cdc.gov/ToxProfiles/tp189.pdf 38091450

[60] WHO. Evaluations of the Joint FAO/WHO Expert Committee on Food Additives (JECFA). 2002; https://apps.who.int/food-additives-contaminants-jecfa-database/Home/Chemical/2698files/1038/2698.html

[61] Jin L , Lynch J, Richardson A, et al. Electronic cigarette solvents, pulmonary irritation, and endothelial dysfunction: role of acetaldehyde and formaldehyde. Am J Physiol Heart Circ Physiol. 2021;320(4):H1510–H1525. 33543686 10.1152/ajpheart.00878.2020 PMC8260384

[62] Lechasseur A , Jubinville E, Routhier J, et al. Exposure to electronic cigarette vapors affects pulmonary and systemic expression of circadian molecular clock genes. Physiol Rep. 2017;5(19):e13440. 29038357 10.14814/phy2.13440 PMC5641932

[63] CIR. Safety Assessment of Glycerin as Used in Cosmetic. 2014.; https://www.cir-safety.org/sites/default/files/glycerin.pdf

[64] Stefaniak AB , Ranpara AC, Virji MA, LeBouf RF. Influence of e-liquid humectants, nicotine, and flavorings on aerosol particle size distribution and implications for modeling respiratory deposition. Front Public Health. 2022;10:782068. 35372219 10.3389/fpubh.2022.782068 PMC8968757

[65] DeVito EE , Krishnan-Sarin S. E-cigarettes: impact of e-liquid components and device characteristics on nicotine exposure. Curr Neuropharmacol. 2018;16(4):438–459. 29046158 10.2174/1570159X15666171016164430 PMC6018193

[66] Gholap VV , Kosmider L, Golshahi L, Halquist MS. Nicotine forms: why and how do they matter in nicotine delivery from electronic cigarettes? Expert Opin Drug Deliv. 2020;17(12):1727–1736. 32842785 10.1080/17425247.2020.1814736 PMC9361466

[67] Li L , Lee ES, Nguyen C, Zhu Y. Effects of propylene glycol, vegetable glycerin, and nicotine on emissions and dynamics of electronic cigarette aerosols. Aerosol Sci Technol. 2020;54(11):1270–1281. 33116348 10.1080/02786826.2020.1771270 PMC7590927

[68] Shao XM , Friedman TC. Pod-mod vs. conventional e-cigarettes: nicotine chemistry, pH, and health effects. J Appl Physiol (1985). 2020;128(4):1056–1058. 31854246 10.1152/japplphysiol.00717.2019 PMC7191502

[69] Leventhal AM , Madden DR, Peraza N, et al. Effect of exposure to e-cigarettes with salt vs free-base nicotine on the appeal and sensory experience of vaping: a randomized clinical trial. JAMA Netw Open. 2021;4(1):e2032757. 33433597 10.1001/jamanetworkopen.2020.32757 PMC7804919

[70] St Helen G , Dempsey DA, Havel CM, Jacob P, 3rd, Benowitz NL. Impact of e-liquid flavors on nicotine intake and pharmacology of e-cigarettes. Drug Alcohol Depend. 2017;178:391–398. 28704768 10.1016/j.drugalcdep.2017.05.042 PMC5565733

[71] Prochaska JJ , Vogel EA, Benowitz N. Nicotine delivery and cigarette equivalents from vaping a JUULpod. Tob Control. 2022;31(e1):e88–e93. 33762429 10.1136/tobaccocontrol-2020-056367 PMC8460696

[72] Talih S , Balhas Z, Salman R, et al. Transport phenomena governing nicotine emissions from electronic cigarettes: model formulation and experimental investigation. Aerosol Sci Technol. 2017;51(1):1–11. 28706340 10.1080/02786826.2016.1257853 PMC5502764

[73] Yan XS , D’Ruiz C. Effects of using electronic cigarettes on nicotine delivery and cardiovascular function in comparison with regular cigarettes. Regul Toxicol Pharmacol. 2015;71(1):24–34. 25460033 10.1016/j.yrtph.2014.11.004

[74] Spindle TR , Talih S, Hiler MM, et al. Effects of electronic cigarette liquid solvents propylene glycol and vegetable glycerin on user nicotine delivery, heart rate, subjective effects, and puff topography. Drug Alcohol Depend. 2018;188:193–199. 29778773 10.1016/j.drugalcdep.2018.03.042 PMC7193252

[75] FDA. Code of Federal Regulation. § 182.60 Synthetic flavoring substances and adjuvants. 2022; https://www.ecfr.gov/current/title-21/chapter-I/subchapter-B/part-182/subpart-A/section-182.60

[76] Korzun T , Munhenzva I, Escobedo JO, Strongin RM. Synthetic food dyes in electronic cigarettes. Dyes Pigm. 2019;160:509–513.

[77] FDA. Certified Color Additives in Food and Possible Association with Attention Deficit Hyperactivity Disorder in Children. 2011; https://foodpoisoningbulletin.com/wp-content/uploads/FAC-Color-Additives-ADHD.pdf

[78] Peiperl MD , Prival MJ, Bell SJ. Determination of combined benzidine in FD&C Yellow No. 6 (Sunset Yellow FCF). Food Chem Toxicol. 1995;33(10):829–839. 7590527 10.1016/0278-6915(95)00051-3

[79] Robinson G. Tartrazine—the story so far. Food Chem Toxicol. 1988;26(1):73–76.

[80] Basáñez T , Majmundar A, Cruz TB, Allem JP, Unger JB. E-cigarettes are being marketed as “vitamin delivery” devices. Am J Public Health. 2019;109(2):194–196. 30649935 10.2105/AJPH.2018.304804 PMC6336045

[81] Hou C-Y. Is vaping vitamins a good idea? 2022; https://thehill.com/changing-america/well-being/medical-advances/3614097-is-vaping-vitamins-a-good-idea/

[82] FDA. FDA takes action to protect consumers from vaping products with unproven health claims. 2021; https://www.fda.gov/consumers/health-fraud-scams/fda-takes-action-protect-consumers-vaping-products-unproven-health-claims

[83] FDA. Warning Letter: Vitamin Vape, Inc. 2021; https://www.fda.gov/inspections-compliance-enforcement-and-criminal-investigations/warning-letters/vitamin-vape-inc-617787-12012021

[84] Roemer E , Schorp MK, Piadé JJ, et al. Scientific assessment of the use of sugars as cigarette tobacco ingredients: a review of published and other publicly available studies. Crit Rev Toxicol. 2012;42(3):244–278. 22263649 10.3109/10408444.2011.650789 PMC3296517

[85] Soussy S , El-Hellani A, Baalbaki R, et al. Detection of 5-hydroxymethylfurfural and furfural in the aerosol of electronic cigarettes. Tob Control. 2016;25(Suppl 2):ii88–ii93. 27798321 10.1136/tobaccocontrol-2016-053220

[86] Rezk-Hanna M , Talhout R, Jordt SE. Sugars and sweeteners in tobacco and nicotine products: FDA regulatory implications. Nicotine Tob Res. 2023;25(4):838–840. 36148496 10.1093/ntr/ntac222 PMC10032193

[87] Rosbrook K , Erythropel HC, DeWinter TM, et al. The effect of sucralose on flavor sweetness in electronic cigarettes varies between delivery devices. PLoS One. 2017;12(10):e0185334. 28968411 10.1371/journal.pone.0185334 PMC5624589

[88] Duell AK , McWhirter KJ, Korzun T, Strongin RM, Peyton DH. Sucralose-enhanced degradation of electronic cigarette liquids during vaping. Chem Res Toxicol. 2019;32(6):1241–1249. 31079450 10.1021/acs.chemrestox.9b00047 PMC9831380

[89] El-Hage R , El-Hellani A, Haddad C, et al. Toxic emissions resulting from sucralose added to electronic cigarette liquids. Aerosol Sci Technol. 2019;53(10):1197–1203. 36506805 10.1080/02786826.2019.1645294 PMC9733909

[90] Erythropel HC , Jabba SV, DeWinter TM, et al. Formation of flavorant-propylene glycol adducts with novel toxicological properties in chemically unstable e-cigarette liquids. Nicotine Tob Res. 2019;21(9):1248–1258. 30335174 10.1093/ntr/nty192 PMC6698951

[91] Kerber PJ , Peyton DH. Kinetics of aldehyde flavorant-acetal formation in e-liquids with different e-cigarette solvents and common additives studied by ^1^H NMR Spectroscopy. Chem Res Toxicol. 2022;35(8):1410–1417. 35830545 10.1021/acs.chemrestox.2c00159 PMC10861150

[92] Larcombe A , Allard S, Pringle P, et al. Chemical analysis of fresh and aged Australian e-cigarette liquids. Med J Aust. 2022;216(1):27–32. 34528266 10.5694/mja2.51280

[93] Vas CA , Porter A, McAdam K. Acetoin is a precursor to diacetyl in e-cigarette liquids. Food Chem Toxicol. 2019;133:110727. 31377138 10.1016/j.fct.2019.110727

[94] Jabba SV , Diaz AN, Erythropel HC, Zimmerman JB, Jordt SE. Chemical adducts of reactive flavor aldehydes formed in e-cigarette liquids are cytotoxic and inhibit mitochondrial function in respiratory epithelial cells. Nicotine Tob Res. 2020;22(Suppl 1):S25–S34. 33320255 10.1093/ntr/ntaa185 PMC8224836

[95] FreemanVapeJuice. What is vape juice? 2023.; https://www.freemanvapejuice.com/pages/vape-juice-flavors. Accessed April 6, 2023.

[96] FDA. Ensuring Compliance with the Tobacco Control Act and Enforcing the Law. 2021; https://www.fda.gov/tobacco-products/about-center-tobacco-products-ctp/ensuring-compliance-tobacco-control-act-and-enforcing-law

[97] Behar RZ , Luo W, McWhirter KJ, Pankow JF, Talbot P. Analytical and toxicological evaluation of flavor chemicals in electronic cigarette refill fluids. Sci Rep. 2018;8(1):8288. 29844439 10.1038/s41598-018-25575-6 PMC5974410

[98] Dinu V , Kilic A, Wang Q, et al. Policy, toxicology and physicochemical considerations on the inhalation of high concentrations of food flavour. NPJ Sci Food. 2020;4:15. 33083547 10.1038/s41538-020-00075-y PMC7541606

[99] Sears CG , Hart JL, Walker KL, Robertson RM. Generally recognized as safe: uncertainty surrounding e-cigarette flavoring safety. Int J Environ Res Public Health. 2017;14(10):1274. 29065549 10.3390/ijerph14101274 PMC5664775

[100] Nayak P , Kemp CB, Redmon P. A qualitative study of vape shop operators’ perceptions of risks and benefits of e-cigarette use and attitude toward their potential regulation by the US Food and Drug Administration, Florida, Georgia, South Carolina, or North Carolina, 2015. Prev Chronic Dis. 2016;13:E68. 27197081 10.5888/pcd13.160071 PMC4877178

