# Supplementary material for: A Review of the Toxicity of Ingredients in e-Cigarettes, Including Those Ingredients Having the FDA’s “Generally Recognized as Safe (GRAS)” Regulatory Status for Use in Food
Source: Nicotine Tob Res. 2024 May 24;26(11):1445–54. doi: 10.1093/ntr/ntae123 (PMC11494494; doi:10.1093/ntr/ntae123)
Supplement: ntae123_suppl_Supplementary_Material [file ntae123_suppl_supplementary_material.docx]

**Supplementary References**

101. FDA. Substances Generally Recognized as Safe. Federal Register, Volume 81, Issue 159 (August 17, 2016). National Archives and Records Administration, 54960–55055. Accessed December 14, 2023, <https://thefederalregister.org/81-FR/Issue-159>

102. FDA. Draft Guidance for Industry: Regulatory Submissions to OFAS, Part VI GRAS Notices. <https://www.fda.gov/regulatory-information/search-fda-guidance-documents/draft-guidance-industry-regulatory-submissions-ofas-part-vi-gras-notices>

103. FDA. GRAS Notices. <https://www.cfsanappsexternal.fda.gov/scripts/fdcc/?set=GRASNotices>

104. FEMA. Flavor Ingredient Library. <https://www.femaflavor.org/flavor-library/search>

105. Woutersen RA, Appelman LM, Van Garderen-Hoetmer A, Feron VJ. Inhalation toxicity of acetaldehyde in rats. III. Carcinogenicity study. *Toxicology*. Oct 1986;41(2):213-31. doi:10.1016/0300-483x(86)90201-5

106. CTP. *Harmful and Potentially Harmful Constituents in Tobacco Products; Established List; Proposed Additions; Request for Comments*. 2019. 08/05/2019. Accessed April 25, 2023. <https://www.federalregister.gov/documents/2019/08/05/2019-16658/harmful-and-potentially-harmful-constituents-in-tobacco-products-established-list-proposed-additions>

107. Behar RZ, Luo W, Lin SC, et al. Distribution, quantification and toxicity of cinnamaldehyde in electronic cigarette refill fluids and aerosols. *Tob Control*. Nov 2016;25(Suppl 2):ii94-ii102. doi:10.1136/tobaccocontrol-2016-053224

108. (2024). NCfBI. PubChem Compound Summary for CID 8857, Ethyl Acetate. . doi:<https://pubchem.ncbi.nlm.nih.gov/compound/Ethyl-Acetate>.

109. (NIOSH) TNIfOSaH. *NIOSH Pocket Guide to Chemical Hazards: Ethyl acetate*. October 30, 2019. <https://www.cdc.gov/niosh/npg/npgd0260.html>

110. Clark GC. Acute inhalation toxicity of eugenol in rats. *Arch Toxicol*. 1988;62(5):381-6. doi:10.1007/bf00293627

111. Blount BC, Karwowski MP, Morel-Espinosa M, et al. Evaluation of Bronchoalveolar Lavage Fluid from Patients in an Outbreak of E-cigarette, or Vaping, Product Use-Associated Lung Injury - 10 States, August-October 2019. *MMWR Morb Mortal Wkly Rep*. Nov 15 2019;68(45):1040-1041. doi:10.15585/mmwr.mm6845e2

112. Bitzer ZT, Goel R, Reilly SM, et al. Effect of flavoring chemicals on free radical formation in electronic cigarette aerosols. *Free Radic Biol Med*. May 20 2018;120:72-79. doi:10.1016/j.freeradbiomed.2018.03.020

113. Christensson JB, Matura M, Gruvberger B, Bruze M, Karlberg AT. Linalool--a significant contact sensitizer after air exposure. *Contact Dermatitis*. Jan 2010;62(1):32-41. doi:10.1111/j.1600-0536.2009.01657.x

114. Morris AM, Leonard SS, Fowles JR, Boots TE, Mnatsakanova A, Attfield KR. Effects of E-Cigarette Flavoring Chemicals on Human Macrophages and Bronchial Epithelial Cells. *Int J Environ Res Public Health*. Oct 22 2021;18(21):11107. doi:10.3390/ijerph182111107

115. Hua M, Omaiye EE, Luo W, McWhirter KJ, Pankow JF, Talbot P. Identification of Cytotoxic Flavor Chemicals in Top-Selling Electronic Cigarette Refill Fluids. *Sci Rep*. Feb 26 2019;9(1):2782. doi:10.1038/s41598-019-38978-w
